# Accessibility for persons with mobility impairments within an informal trading site: A case study on the markets of Warwick, South Africa

**DOI:** 10.4102/ajod.v3i1.120

**Published:** 2014-11-21

**Authors:** Pragashnie Naidoo, Helga Elke Koch, Jassmine Anderson, Prashika Ghela, Perusha Govender, Nausheena Hoosen, Halima Khan

**Affiliations:** 1 Occupational Therapy Department, University of KwaZulu-Natal, South Africa

## Abstract

**Background:**

There are a number of informal trading sites across cities in sub-Saharan Africa, of which the markets of Warwick is one example. Since the informal economy is an important contributor to a city’s economy as well as a source of employment, it is important for these sites to be accessible for all persons. Whilst the South African government has put structures in place to identify and remove environmental barriers in order to meet the individual needs of persons with mobility impairments and improve their quality of life, persons with mobility impairments still face barriers and restricting environments that prevent them from participating in society and its social and economic activities.

**Objectives:**

This case study aimed at exploring accessibility within the markets of Warwick for persons with mobility impairments by an ergonomic assessment, augmented by voices of participants within the market.

**Method:**

A qualitative, instrumental, single case study design was utilised with purposive sampling of the markets of Warwick as the study setting. Multiple sources of data were gathered, such as semi-structured interviews, direct observations of an environmental survey supported by photographs, and the authors’ review of relevant documents. Transcriptions were analysed using NVivo 10 software programme with inductive coding.

**Results:**

Whilst policies have been in place since 1996 to adjust infrastructure, the markets of Warwick still remain inaccessible to persons with mobility impairments and do not meet the standardised infrastructural design.

**Conclusion:**

The findings of this study may offer a significant understanding of the complexity of accessibility within an informal trading site and create an awareness of the limitations this has for persons with mobility impairments. Additionally, these findings may assist in effecting a positive change in terms of the infrastructure of the Markets and in continuous advocating for the rights of persons with all disabilities.

## Introduction

Twenty years into South Africa’s democracy, there has been a significant improvement in recognising the Rights of Persons with Disabilities. Policies and legislation include the Constitution, the Integrated National Disability Strategy (INDS) of 1997, *Promotion of Equality and Prevention of Unfair Discrimination Act* (PEPUDA, or the *Equality Act No. 4* of 2000) and the *Employment Equity Act* (EEA), *No. 55 of 1998*. South Africa was also one of the countries to sign the treaty that emerged from the United Nations (UN) Convention on the Rights of Persons with Disabilities, thereby agreeing to ensure that individuals who have mobility impairments have access on an equal basis with others, to the physical environment, transportation, information and communications – including information and communications technologies and systems and other facilities and services open or provided to the public, both in urban and in rural areas (United Nations General Assembly 2006).

The Disability Policy Guideline of South Africa offers standards to making public buildings accessible and allows persons with disabilities to redeem the benefits from the services provided by the government (Department of Public Works [DoPW] 2010:10). Attention is directed to improving accessibility of ramps, parking and ambulation facilities. Since then, the standards have been revised for the South African Bureau of Standard’s Code of Practice: The Application of the National Building Regulations (South African National Standard [SANS] 2011) to further promote accessibility for persons with disabilities (Part S: Facilities for persons with disabilities). Importantly, the latest revision occurred in 2011, following which the authors have been unable to locate recent literature regarding compliance of these aspects to ensure accessibility. Prior to this, Maritz noted that there was substantial non-compliance of the relevant aspects of the Code of Good Practice to ensure accessibility. Reasons for this were that the existing built environment was developed in compliance with previous building laws, the regulatory system is inadequate and difficult to enforce, and the existing legislation at the time was in conflict with the South African Constitution, giving the right to barrier-free access for all (Maritz 2008).

Anecdotal evidence exists where improvements to accessibility have occurred within the formal structures related to recent developments and where there is access to funding opportunities within the public and private sector. There is no evidence locally, and within Africa, related to the investigation of accessibility of informal trading sites which play a significant role in the country’s economic, social and cultural activities. Indeed, informal markets are both a resource for persons with disabilities to generate income through informal employment opportunities, as well as providing access to essential goods and services.

The markets of Warwick (hereafter referred to as ‘the markets’) were chosen as a case study to obtain evidence on the accessibility of one such site. This is specifically related to whether the infrastructural design adequately supports persons with mobility impairments by meeting the South African standardised requirements as well as exploring the perceptions of those persons with mobility impairments with respect to occupational safety factors within the markets.

Warwick Junction, located in the centre of Durban, South Africa, is considered to be ‘the site of the most informal trading in the eThekwini municipality’ (Hemson 2003:3). Daily, hundreds of commuters, traders and visitors enter the nine diverse markets by taxis, buses and trains. The markets serve mostly people living in rural areas, local and surrounding townships and informal settlements in the province, as it is the main transport hub for traders and commuters who visit the area to trade in goods and services. Most of the traders are from outside Durban city, ‘trading and maintaining links to the countryside in a circulation of people and commodities which makes the survival of the rural and urban poor possible’ (Hemson 2003:6).

The markets, established in 1910 along the sidewalks of Durban’s Victoria Street, have become an important heritage for the people of Durban. There are approximately 8000 traders who operate within Warwick Junction. Currently, many traders within the markets are third and fourth generation descendants of original traders (Skinner 2009:106). Through the challenges of poverty and trials of apartheid, this culturally rich architecture has become an important contributor to the South African economy.

When visiting the markets, one will experience the unforgettable sights and sounds of the sales of different goods and services. Under the unfinished flyovers, ad hoc bridges and bus shelters, the markets offer a place of trading and occupations that are rarely found anywhere else in the city. Here, the commuters can receive advice and purchase products from traditional herbalists, handcrafted beaded jewellery, livestock, fruit and vegetables, bovine head meat and even shebeens^1^, which serve thousands of people entering and leaving the markets daily.

The authors explored the markets by conducting interviews with traders and porters (barrow operators). An environmental analysis assisted in placing their views and opinions within their context and the perusal of relevant documents and policies allowed the authors to frame the outcomes.

## Research method and design

### Design

A qualitative approach using an instrumental single case study design with multiple sources of data was used (Creswell 2013; Yin 2014). The study setting and participants were selected through purposive sampling (Table 1).

**TABLE 1:** Overview of methods and participants.

Participant(s) and sampling method	Gender or age	Diagnosis	Duration of interview	Location of data collection	Process	Inclusion criteria	Exclusion criteria
Trader 1 Themba^*^ (Purposive)	Male 38 years	Left Cerebrovascular accident, (CVA), resultant right hemiplegia	±60 minutes	Early morning market (at the participants’ stalls)	Digitally voice and video recorded	Either gender Age 18–80 Mobility impairment	Participants with any form of psychiatric condition
Trader 2 Sihle^*^ (Purposive)	Male 52 years	Arthritis affecting lower limbs	±60 minutes	-	-	(including wheelchair users)	Participants with hearing or visual impairments
Passer-by 1 Moses^*^ (Purposive)	Male 27 years	Paraplegia	±60 minutes	Early morning market (near the taxi rank that the participant accessed)	-	Traders that trade within one of the 9 markets	-
Porter 1 Philip^*^ (Purposive)	Male 50 years	Congenital foot deformity	±60 minutes	Brook street market (within the participant’s immediate working environment)	-	-	-
Co-founder 1: NGO (Asiye eTafuleni) Patrick (Purposive)	Male 59 years	N/A	±60 minutes	Asiye eTafuleni offices	Digitally voice and video recorded	Individual who is directly involved and operational within the markets	
Co-founder 2: NGO (Asiye eTafuleni) Richard (Purposive)	Male 54 years	-	-	-	-	-	-

* Note: Pseudonyms used.

NGO, non-governmental organization; N/A, not applicable.

### Study setting

**FIGURE 1:**
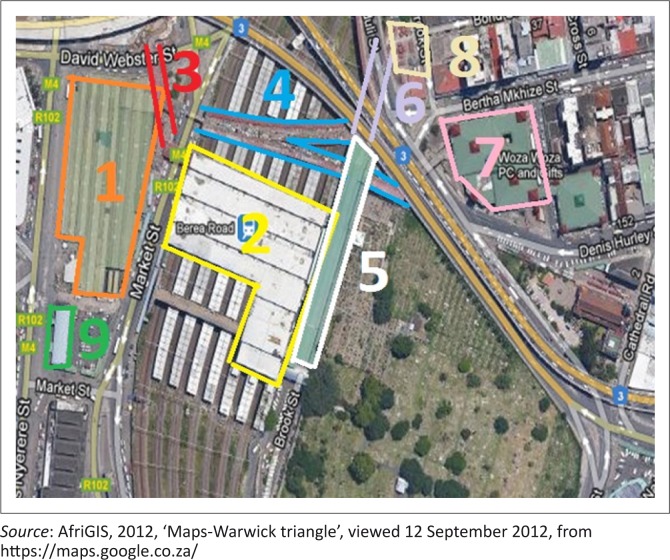
Aerial view of the nine markets of Warwick (1) Early Morning market; (2) Berea Station mart; (3) Music Bridge; (4) Herb market; (5) Brook Street market; (6) Lime and Impheph market; (7) Victoria Street market; (8) Bead market; (9) Bovine Head market.

Figure 1 provides an aerial view of the markets.

This informal market is made up of nine smaller to medium-sized markets. When visiting the markets, one can make use of a main railway station, five bus terminals and 19 taxi ranks. Roads, walkways and pedestrian bridges intersect the area, which is 10 minutes from the city centre. The markets offer a wide variety of facilities, services and merchandise. Some of the goods and services that can be found in the markets include traditional cuisine, fresh produce, poultry, spices, flowers, traditional medicine, beadwork, traditional arts, crafts, music and entertainment merchandise, clothing and accessories (Dobson, Skinner & Nicholson 2009:5).

### Procedure

Data was gathered at the markets over a period of a week.

**Semi-structured interviews**: with six participants with open-ended questions were conducted. The central question was related to the participants’ experiences within the markets, with prompts towards barriers and facilitators and issues surrounding accessibility. Interviewees comprised two traders, one passer-by and one porter – all with a mobility impairment – and two co-founders of a non-governmental organisation (Asiye eTafuleni 2013) that operates within the markets. These demographics are represented in Table 1. The difficulty in obtaining participants with mobility impairments who utilised the markets is an indication of the inaccessibility of the area for them.

**Direct observation**: in this study included an environmental (accessibility) survey. Direct observation in a case study occurs when researchers visit the location or study site in order to gather data. The data gathered can be in the form of observations that could be formal or casual. Yin (2014) suggests that using multiple observers ensures the reliability of the observations made. Data gathered and observations made by the authors are supported by visual aids (photographs), for which ethical clearance was obtained.

Additionally, authors reviewed various national and international *documents* such as policies, acts, briefs, legislation, regulations guidelines related to accessibility, disability and trading within informal markets – and perused relevant audiovisual material on the markets.

### Analysis

Semi-structured interviews were digitally voice and video recorded. The accessibility (environmental) survey was supported with visual aids (photographs) and measurements. To ensure reliability, the authors collaborated with an isiZulu translator who assisted in verifying the transcriptions and translations. The authors used NVivo 10 (qualitative data analysis software) with inductive reasoning during the coding process (Miles, Huberman & Saldaña 2014). Specific types of coding that were used in this study included initial coding (in-vivo and process coding); descriptive coding; emotion coding and values coding (Saldaña 2013). Using information gained, *inter alia* through interview transcriptions and data from the environmental analysis, the results were pooled and common themes were merged in order to facilitate a clear and concise discussion.

#### Ethical considerations

Issues surrounding informed consent, confidentiality, beneficence, veracity and scientific honesty were observed throughout the research process. Ethical approval was obtained from the various gatekeepers of the study through an ethical process review as well as from participants themselves (informed consent). Confidentiality was ensured by participants being informed of the potential use of the data obtained; video-recordings were viewed only by the authors and measures for confidential data storage were followed. Pseudonyms have been used during all methods of dissemination of the research data. The principles of beneficence and non-maleficence were upheld by being sensitive to non-verbal behaviours of participants, by allowing withdrawal of participation if this was necessary, and by the non-invasive nature of the questions in the interviews. Veracity was ensured by adequate acknowledgement of sources of data, documentation and keeping of accurate records, by reviews of transcriptions against audio and audiovisual recordings and by being explicit about the aims of the study. Scientific honesty as outlined in the Singapore Statement on Research Integrity (2010) was also considered in the reporting and dissemination of the data and research findings.

## Trustworthiness and rigour

A number of measures were implemented to ensure the trustworthiness of this study. These included, *inter alia*, credibility, dependability, confirmability and reflexivity.

Various techniques to ensure *credibility* or *authenticity* (Brink, Van der Walt & Van Rensburg 2012) of the findings were employed. These included the use of multiple sources of data with data triangulation (Olsen 2004; Yin 2014), for example, documents, direct observation through the accessibility survey and interviews, peer debriefing (in order to probe the biases that may have affected the study), as well as member checking (where the interpretation of the data was reviewed and verified by the participants).

**Dependability** was ensured through the reduction of researcher bias through investigator triangulation.

**Confirmability** is said to guarantee that the findings, conclusions and recommendations are supported by the data with internal agreement between the investigators’ interpretation and the actual evidence (Brink *et al*. 2012). This was ensured during the accessibility survey in which actual measurements were taken and matched against standard measurement data (Ormerod & Newton 2003; SANS 10400-S 2011). Various policies as well as the lived experiences captured by way of the semi-structured interviews were used together with these findings in order to move towards the internal agreement that represents confirmability and overall trustworthiness of the study.

## Findings

**FIGURE 2:**
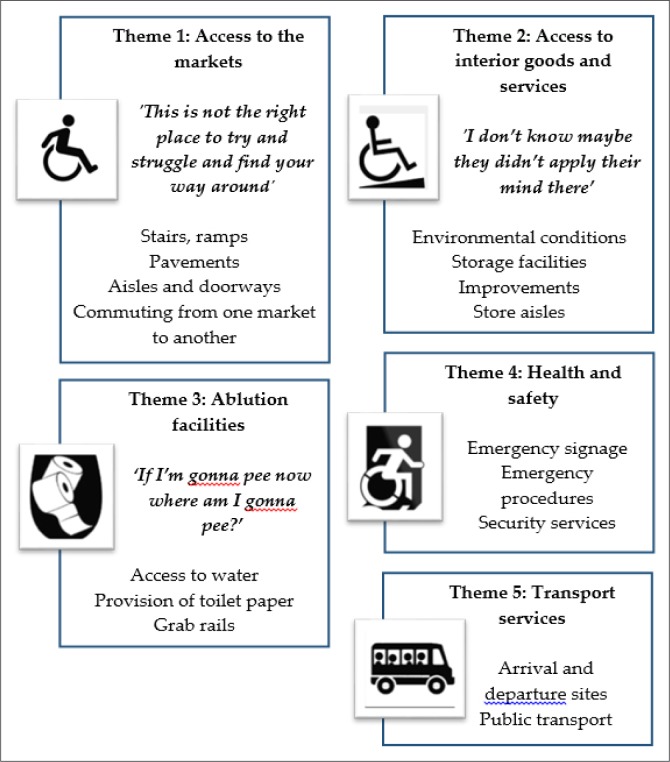
Themes emerging from the study.

As seen previously in Figure 1, the markets are surrounded by staircases, foot-bridges, main roads and ramps leading to the entrances. Findings from the environmental accessibility survey and interviews enhanced the authors’ understanding of the barriers and challenges faced by persons with mobility impairments within the markets. Five major emerging themes will be discussed in this article. These are summarised in Figure 2.

### Theme 1: Access to the markets (‘*This is not the right place to try and struggle to find your way around*’)

**FIGURE 3:**
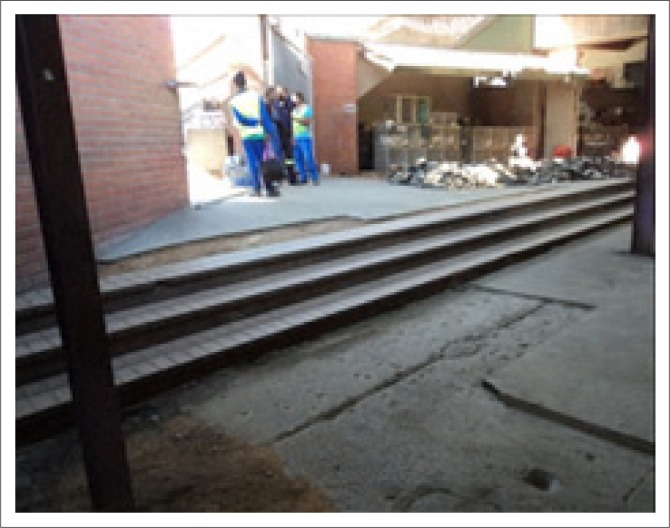
Entrance with no handrails on either side of the staircase.

The environmental survey raised various issues related to access points (such as roads, staircases, bridges, ramps and sidewalks) to the nine markets. These included the quality of the ramps and stairs (including handrails), sidewalks and road surfaces leading to these access points. Ramps and certain stairs failed to meet the standard safety requirements (Figure 3).

Additionally, floor finishes did not satisfy design specifications (unsmooth and slippery), therefore posing an increased risk for and fear of falls for persons with mobility impairments (Ormerod & Newton 2003:145):

‘So I battle you know if I go up the steps … uh … it’s better if there are rails then I hold against the rails and walk up or down the steps’. (Themba)

‘I don’t even use ramps because I’m afraid I might lose balance so I don’t ever use them’. (Philip)

**FIGURE 4:**
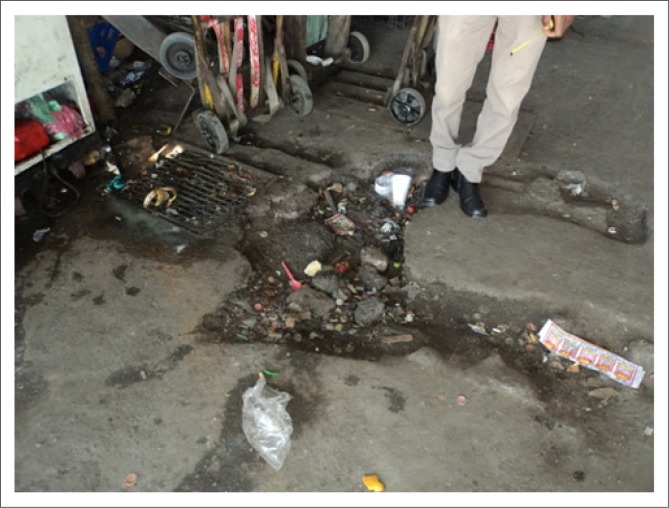
Pothole (120 mm deep and 530 mm wide).

Doorways exceed the standard requirement, namely 750 mm width (SANS 10400-S 2011: section 4.6) and participants, therefore, reported no difficulties moving through the doorways and entrances to the markets. Surrounding sidewalks leading to access points were in a poor state, with many potholes and uneven floor surfaces being noted (Figure 4).

**FIGURE 5:**
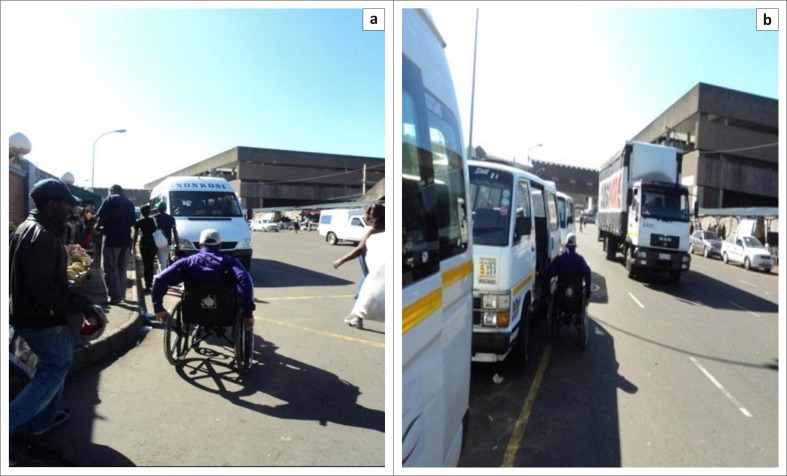
Wheelchair user accessing a taxi from the road.

Potholes were often filled with litter and broken glass, posing health and safety risks for persons using wheelchairs. Whilst several sidewalks leading to access points met the design specification of 1500 mm in width, some traders occupied most of the sidewalk space to display their goods. This inevitably leaves limited space for persons with mobility impairments, especially those utilising wheelchairs, as there is no alternative accessible and safe route(SANS 10400-S 2011: section 4.4). These poorly conditioned sidewalk surfaces and limited spaces often result in wheelchair users mobilising on the road to access the markets and/or taxi services (Figure 5). ‘The road surface I would say, there is some work that needs to be done …, because there’s no, like, the pavement for us’ (Moses).

Since the roads surrounding the markets and those leading to access points are main roads, they have a constant heavy flow of traffic. There are thus serious safety risks associated with the use of the surrounding roads to access the markets for persons with mobility impairments.

‘Pavements are narrow … and also some taxis … they [drive] on the wrong side of the road. People have to jump from … the side of moving [in the road] or … on the high, they are on … if I’m … they are on the fast lane …, you know, people are risking their life accessing taxis from the fast lane’. (Patrick)

### Theme 2: Access to interior goods and services (‘I don’t know, maybe they didn’t apply their mind there’)

Store aisles and passageways within the various markets satisfy the design specifications. However, participants reported difficulties moving in this space because of congested and busy aisles. ‘They are not wide enough, but considering the number of people, you know, they cannot be enough because of the number of porters and consumers’ (Philip).

Many traders utilise the services of porters to transport their goods to and from their stalls and storage facilities. The environmental survey revealed two storage facilities. The entrances into the one market storage facility appeared poorly designed as a result of the steep ramps, which did not meet design specifications. A participant reported the following:

‘Uhm … [*chuckles*] there are ramps, uh there are you know, they make storage uh, you know, accessible uh except one in Brook Street uhm … the city I didn’t, I don’t know, maybe they didn’t apply their mind there, it’s very steep …‘ (Patrick)

As a result, traders and porters no longer utilise the storage facility located outside this market to store their goods. Transportation of goods up the steep ramps poses a risk and is considered difficult to achieve.

‘We transport goods from their trading sites into storages. Some storages we have to climb the steps carrying goods on both shoulders [*points to shoulders*]. It’s very, very difficult because you can even fall and damage your customer’s goods or even injure yourself [*points overhead – describing stairs*]’. (Philip)

**FIGURE 6:**
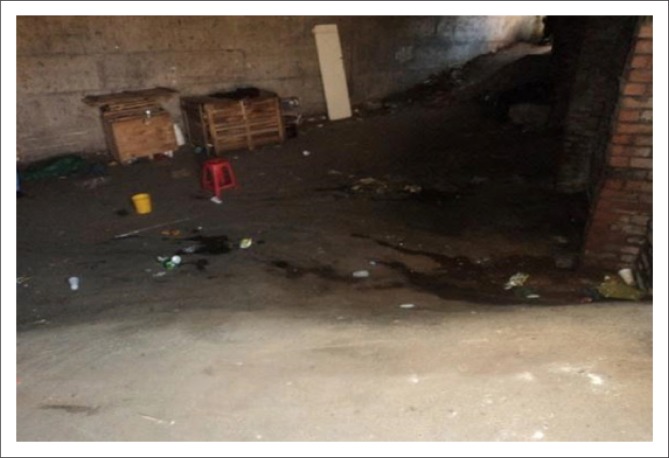
Abandoned storage facility located outside the bead market utilised as an ablution facility.

Subsequently, this storage facility has been abandoned, and is now used as an ablution facility, which poses a health risk to traders and consumers (Figure 6).

This study has also highlighted additional access issues. It is a common occurrence for persons with mobility impairments to be carried up the stairs at one of the markets in order to access stalls or the train station located within the market.

‘Well if you here [*on one side of the railway line*] and you disabled there’s no way you can cross the railway line, that’s absolutely clear, so you will have to get someone to carry you up all the stairs uh … and once you’ve managed that then you [*get*] through [*the building going*] over the station, then you’ve got to fall down the other side. So the only way you can cross would be to go through the Herb market which still … is got a huge flight of stairs uh … if you came up these stairs into the music bridge it might be a little bit easier uh … but no, it’s getting from this side of town to Grey street [*the street on the other wide of the railway line*] is impossible unaided’. (Richard)

Persons with mobility impairments are unable to access upper-level markets. One of the markets is only accessible by way of three flights of staircases comprising of 36 stairs. Staircases throughout the markets did not satisfy the standard requirements. Most staircases are in a poor condition, with damaged, uneven surfaces, with variability being noted in the heights of the steps. This poses a safety (tripping) hazard:

‘I experience a lot of difficulty especially when walking up stairs my body gets sore in such a way I feel like crawling up, you know, when I go up the stairs’. (Sihle)

Ramps throughout the markets do not satisfy the design specifications as there is an absence of handrails in some instances, whilst in other areas some are too steep for use and floor surfaces are not smooth, making access to markets, therefore, difficult for persons with mobility impairments.

The environmental survey revealed that floor finishes throughout the markets were in a poor condition. Most of the floors did not satisfy design specifications (smooth and non-slip flooring), therefore posing a safety hazard for persons with mobility impairments. ‘I would say it’s not that easy, because there are potholes, but I’m used to the area’ (Themba).

### Theme 3: Ablution facilities (‘If I’m gonna pee now where am I gonna pee?’)

Accessible ablution facilities include access to toilet cubicles, toilets, washbasins as well as other equipment, which can be utilised by persons in wheelchairs or other persons with mobility impairments. Currently there are no accessible ablution facilities for persons with mobility impairments within the markets.

‘There are no disabled toilets around here, if I’m gonna pee now where am I gonna pee? If maybe they could put … disabled toilets around the markets, so it would make my life easier’. (Moses)

Public ablution facilities are available. However, they are not wheelchair accessible, nor meet the standard criteria for use by persons with disabilities. The absence of these facilities may therefore prevent persons with mobility impairments from accessing the markets.

**FIGURE 7:**
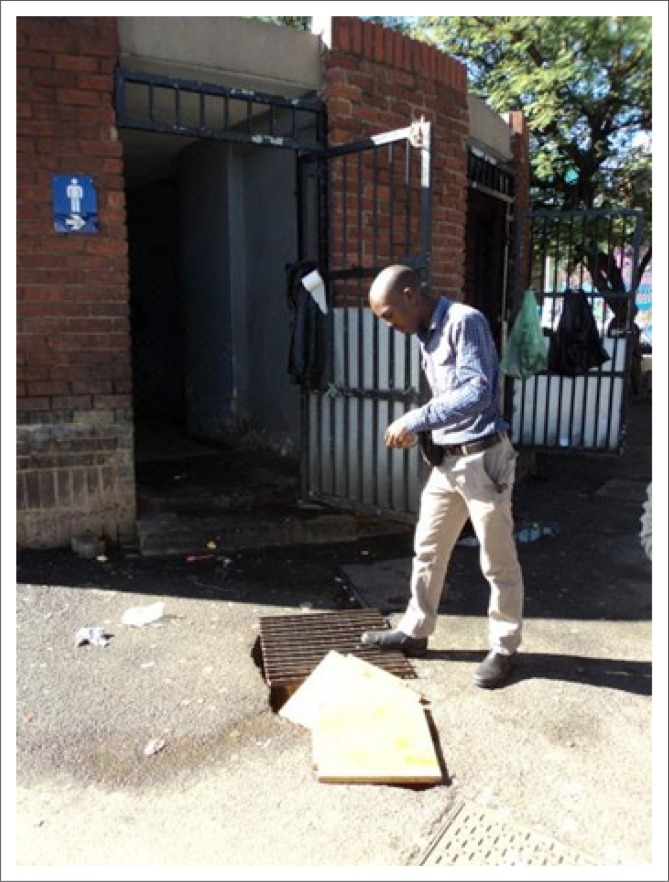
Entrance of public ablution facility with large pothole covered with wood, and absence of ramp leading into ablution facility.

The environmental survey revealed poor terrain for mobilisation (for example, open manholes and large potholes), presenting difficulties and potential danger for those wanting to access the facilities (Figure 7).

**FIGURE 8:**
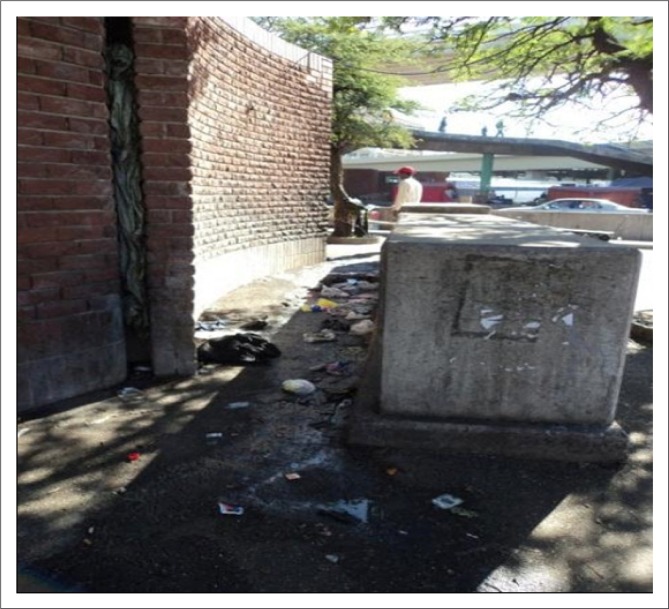
Area used as ablution facility when public ablution facilities are closed after 16:00 daily.

The entrance to the ablution facility does not have a ramp; it, however, does have steps and therefore access for those persons with mobility impairments is hindered. Moreover, the ablution facilities surrounding the markets are not accessible to consumers after 16:00. Although this is considered ‘rush hour’ with many persons passing through the area (as a thoroughfare) after their working day, these facilities remain locked. As a consequence, persons needing to use the ablution facilities, utilise public stall areas as an ‘ablution facility’ (Figure 8).

The stall situated behind the ablution facilities in two of the markets has, hence, become a severe health risk because of urine and faeces on sidewalks which run into the surrounding drains.

### Theme 4: Health and safety

**FIGURE 9:**
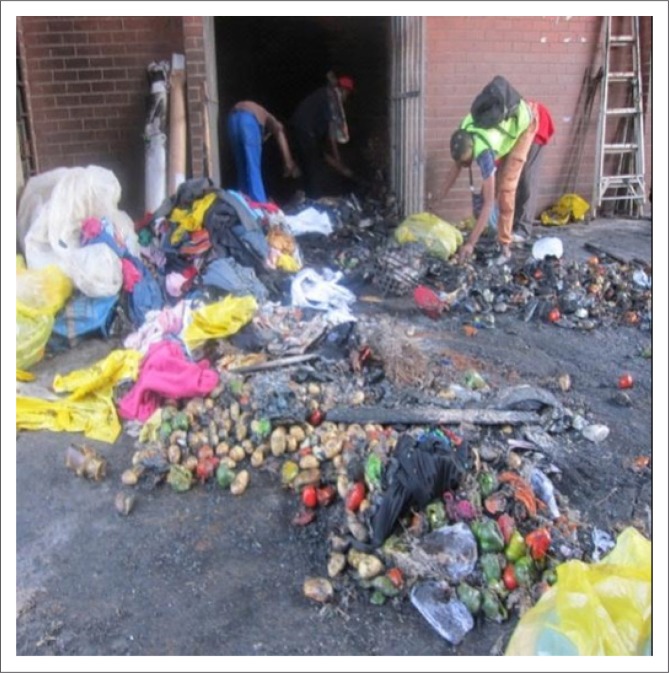
Recent fire damage resulting in loss of trader goods and damage to storage facilities.

Health and safety issues were found to be a significant problem within the nine markets. Firstly, emergency signage is located within only two of the nine markets. Secondly, there is no indication as to where the emergency exits are situated. Thirdly, participants were unaware of the procedures to follow in an emergency situation. Lastly, as already mentioned, severe health risks flow from utilising certain areas for ablution which were not designated for ablution use. In the extreme and unfortunate case of a fire or an emergency requiring fast exit from the markets, factors one to three will reduce the ability of persons with mobility impairments utilising the markets to exit buildings (Figure 9).

Security services, such as guards, are available within six of the nine markets. Traders within all nine of the markets belong to a crime prevention team in order to prevent and reduce the crime within the markets. These are known as the ‘Traders against Crime’ (TAC) and the ‘Community Policing Forum’ (CPF). This results in adequate and sufficient security within the markets and also reduces the frequency and occurrences of crime. According to the participants interviewed, all felt safe and secure within the markets as they are aware of the security available.

### Theme 5: Transport services

Participants reported difficulties when crossing the roads that surround the markets. The difficulty of this task varied from participant to participant, and was dependent on their level of physical disability. A participant reported the following:

‘Ja, it is a bit of a challenge, some other taxi drivers you know they don’t want to wait for me to like to get across you know, so others they are a bit rude’. (Moses)

**FIGURE 10:**
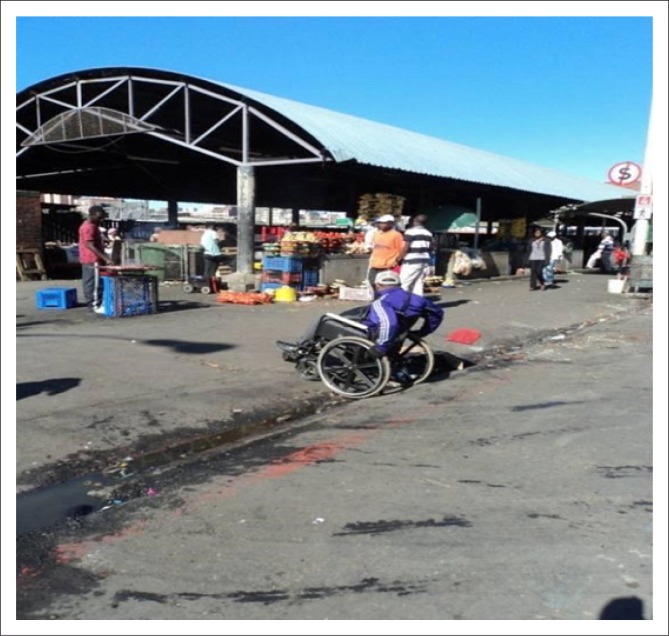
Wheelchair user observed crossing Market Avenue from pick-up zone located on opposite side of the road.

Participants also appeared to be unfamiliar with the context of designated disability parking bays within and surrounding the markets. The environmental survey revealed parking facilities available and accessible to only three of the nine markets, two of which have parking facilities available for both traders and consumers. There are a number of taxi pick-up zones which are located around the nine markets with each taxi zone servicing different geographical areas. According to the participants, they travel to the markets by public transport, more specifically taxis, and arrive and depart in close proximity to the market area (Figure 10).

The one market taxi drop-off zone adhered to specifications and standards by having clearly marked mini-bus road markings. The designated taxi parking spaces are also accessible from the drop-off to the market. However, alongside the taxi pick-up zones are kerbs which lead onto sidewalks which do not have ramps from the road surface onto the sidewalks. The height of the kerbs measure approximately 170 mm which exceeds the standard requirement of 100 mm (Ormerod & Newton 2003:43). Thus, persons using wheelchairs are obliged to propel themselves on the road when needing to access the taxis independently. Otherwise, most will require assistance from others to transfer from sidewalks onto the road surface.

For four of the markets, there are no allocated taxi pick-up zones. Additionally, persons with mobility impairments will not be able to access the taxi pick-up zone of one market, because of the stairs linked to the over-riding bridge.

There is one main bus rank located in one of the markets which accommodates approximately 25 buses.

A train station’s platform leads to the markets over staircases. There are no accessible ramps available. This, therefore, hinders persons with mobility impairments in accessing the station and the markets.

## Discussion

The authors explored accessibility within the markets, by gaining the perceptions of persons with mobility impairments within the markets, supported by direct observations through an environmental survey. In order to best represent the findings of this study, the discussion is presented within the context of available literature and policies.

The South African Constitution (1996) declares the founding values of our society to be the achievement of equality, human dignity, and the advancement of human rights and freedoms. The attainment of a good quality of life for persons with disabilities is included in the country’s main objectives, reflecting the systematic integration of persons with disabilities into all policies, plans, programmes and strategies at every level within the sectors and institutions of government (DoPW 2010:3). However, whilst the South African government has put structures in place to identify and remove environmental barriers in order to meet the individual needs of persons with disabilities, barriers and restricting environments remain.

The Application of the National Building Regulations (SANS 010400 2011 Part S: Facilities for persons with disabilities) clearly outlines the necessary technical requirements for the built environment to ensure accessibility for persons with disabilities. The findings of this study have highlighted many violations in the infrastructural design of the markets that pose a health and safety risk for persons with mobility impairments as well as seriously limit their participation and performance in everyday activities.

With respect to accessibility, the environmental survey raised various matters related to access points, namely roads, staircases, bridges, ramps and sidewalks, to the nine markets. Whilst doorways met the specifications by exceeding the standard requirement, the quality of ramps and stairs, sidewalks and road surfaces was problematic. As stated in a discussion paper by the South African Revenue Services (SARS) with reference to persons with disabilities, modifications to infrastructure need to be made in order for specific structures to be accessible for persons with mobility impairments so that they are able to function or perform daily activities. These modifications include the installation of power-operated stairs or lift facilities or guided chairs to be used in a stairway (SARS 2009:12). However, there is an absence of lifts within the markets even in those with multiple levels, with only access by way of steep ramps or staircases. Furthermore, ramps and certain staircases failed to meet standard requirements, thereby preventing persons with mobility impairments from accessing goods from upper levels, with traders who have mobility impairments being limited to trading on lower levels. To move from one side of the markets to the other side divided by the railway line, is impossible without the assistance of another person, who would be willing to carry the wheelchair user and their wheelchair up and down the stairs. Additionally, floor finishes did not comply with specifications, thereby posing a fall risk for those persons with mobility impairments as well as safety for wheelchair users. This therefore is a violation of the rights of persons with disabilities in accessing public facilities.

Storage facilities are in poor condition as ramps are too high and steep, and often used as ablution facilities. The ablution facilities are inadequate and do not cater for persons with mobility impairments as there are no designated accessible toilets, and an absence of grab rails within the toilets.

Roads surrounding the markets and leading to access points have a constant heavy flow of traffic. Local municipalities have the responsibility to provide public transport in an accessible manner to persons with disabilities. According to Ormerod and Newton (2003) taxi ranks should ideally be located adjacent to major attractions such as retail areas, places of employment and entertainment and leisure centres. They further state that ranks should be located within 50 m – 100 m of the facility being served and if this is not possible then seating should be provided at the rank. Where taxi ranks are arranged on the offside of the road, a pick-up and drop-off point nearby on the opposite side should be identified for passengers utilising wheelchairs (Ormerod & Newton 2003:27).

The findings of this study reflected a number of pick-up zones; however only one market adhered to specifications and standards by having clearly marked mini-bus road markings. Whilst public transport services are adequate as they drop-off passengers within close proximity to the market area, taxi ranks are in a poor condition. Although sidewalks exceed the standard width requirement, the available width is less than the standard requirement when they are occupied by traders displaying their goods. Whilst some of this trading on the sidewalks is regulated, there are other areas where it is not. Town planners and city officials have to be aware in their design to accommodate for this informal trading.

The height of kerbs exceeds the standard requirement posing a risk for persons with mobility impairments, especially those using wheelchairs, as they are obligated to propel themselves on the road. The train station was also found to be inaccessible; hence persons with mobility impairments are restricted to taxis and buses as a mode of transport.

For buildings with parking of more than 50 motor vehicles, there must be at least one parking space per 25 parking spaces which is specifically provided for persons with disabilities, and must be clearly demarcated for such use (SANS 10400-S 2011). Therefore those who travel by private transport, for example a car, will experience difficulty accessing parking spaces which are suitable for their needs and may avoid visiting the markets.

There is a lack of emergency signage throughout the markets, therefore commuters, traders and passers-by are unaware of emergency procedures to follow in the unfortunate case of an emergency. There were reports of a fire that occurred recently in the poultry section of one of the markets which destroyed everything. This emphasises the need for measures related to health and safety.

So, whilst constitutions, policies and conventions, internationally and locally, are working towards a common goal of improving accessibility of any public facility, which includes informal trading areas such as the markets, many persons with disabilities continue to face challenges of accessibility (DoPW 2010; South Africa 1996; United Nations General Assembly 2006). This article highlights that implementation of the policies can take significant time and that context-driven solutions should be sought for information trading sites, since they form such a unique yet vital aspect of the country’s economy and access to people’s livelihoods.

## Conclusion

Whilst policies have been in place since 1996 to adjust infrastructure, the markets still remain inaccessible to persons with mobility impairments and do not meet the standardised infrastructural design. Additionally, the general population utilising the markets of Warwick will also be at risk in terms of safety. Findings of this study may be invaluable in effecting a positive change in terms of the infrastructure of informal trading sites and improving accessibility for persons with mobility impairments. Since informal trading sites are a significant contributor to the informal economy throughout Africa, they are an important source of employment opportunity for all persons with disabilities. Additionally, markets are often centralised together with transport facilities, which are important for persons with mobility impairments to access other forms of employment.

A policy brief was submitted to the municipality for these infrastructural changes to be considered in the maintenance of the markets. This is only one step towards advocating for improvements for the people using this cultural heritage site. The lessons learnt may also be valuable in advocating for policy implementation in informal trading sites throughout Africa.

## Footnotes

1 An illicit bar or club where extensive alcoholic beverages are sold.
